# Simultaneous analysis of miRNA-mRNA in human meningiomas by integrating transcriptome: A relationship between PTX3 and miR-29c

**DOI:** 10.1186/s12885-017-3198-4

**Published:** 2017-03-21

**Authors:** Altay Burak Dalan, Sukru Gulluoglu, Emre Can Tuysuz, Aysegul Kuskucu, Cumhur Kaan Yaltirik, Oguz Ozturk, Ugur Ture, Omer Faruk Bayrak

**Affiliations:** 1 Department of Biochemistry, Yeditepe University Medical School, Istanbul, Turkey; 2 Department of Medical Genetics, Yeditepe University Medical School, Istanbul, Turkey; 3 Department of Biotechnology, Institute of Science, Yeditepe University, Istanbul, Turkey; 4 Department of Neurosurgery, Yeditepe University Medical School, Istanbul, Turkey; 5 Department of Molecular Medicine, Capa School of Medicine, Istanbul University, Istanbul, Turkey; 6 Yeditepe Universitesi Hastanesi Genetik Tani Merkezi, Koftuncu Sokak Acıbadem mahallesi Istek Vakfi 3. Kat 34718 No: 57/1, Kadikoy, Istanbul, Turkey

**Keywords:** meningioma, microarray, miRNA, transcriptome, PTX3, miR-29c

## Abstract

**Background:**

Although meningioma is a common disease, there is a lack of understanding of the underlying molecular mechanisms behind its initiation and progression. We used combined miRNA-mRNA transcriptome analysis to discover dysregulated genes and networks in meningiomas.

**Methods:**

Fourteen fresh-frozen meningioma samples and one human meningeal cell line were analyzed by using miRNA and whole transcriptome microarray chips. Data was filtered and analyzed. Candidate miRNAs and mRNAs were selected for validation in fifty-eight patient samples. miRNA and target mRNA relationships were assessed by inhibiting miRNA in meningioma cells. Apoptosis and viability assays were also used as functional tests.

**Results:**

With the whole transcriptome microarray, 3753 genes were found to be dysregulated, and 891 miRNAs were found to be dysregulated as a result of miRNA microarray. Results were combined and analyzed with bioinformatics tools. Top differential pathways included those of inflammation, cancer, and cellular growth and survival. The oncosupressor PTX3 was constitutively low in meningioma samples. Moreover, PTX3 negatively correlated with miR-29c in our samples. Inhibiting miR-29c upregulated the PTX3 level, induced apoptosis of meningioma cells, and decreased cell viability. CABIN1, miR-29c, TMOD1, PTX3, RPL22, SPARCL1 and RELA were correlated with clinicopathological features in patient samples.

**Conclusions:**

Our results present the first integrated mRNA-miRNA analysis in meningiomas. miR-29c-3p and PTX3 are inversely correlated in tissues and meningioma cells, hinting that PTX3 can be regulated by miR-29c-3p. Furthermore, we determined potential clinicopathological markers.

**Electronic supplementary material:**

The online version of this article (doi:10.1186/s12885-017-3198-4) contains supplementary material, which is available to authorized users.

## Background

Meningiomas account for 30% of primary brain tumors and occur at a rate of 5 per 100,000 individuals [1]. They originate from cap cells of the arachnoidal membrane [2], and the peak age for occurrence is the seventh decade of life [3]. Meningiomas are generally benign but malignant meningiomas have a high tendency to recur. First choice of treatment is surgery, and predictive biomarkers for meningioma progression that could guide oncologists for treatment alternatives are insufficient.

Although meningiomas are common, there is a lack of understanding of underlying molecular mechanisms behind their initiation and progression. To elucidate some of these mechanisms, we used combined miRNA-mRNA transcriptome analysis to discover novel genes and networks in meningiomas. 14 fresh-frozen meningioma samples were used to integrate miRNA and mRNA microarray analysis. Herein, we describe integrated analysis of gene networks that might play an important role in the initiation and progression of meningiomas, with emphasis on the downregulation of tumor suppressor PTX3 via miR-29c.

## Methods

### Sample collection

Fifty-eight fresh samples of meningioma tumor tissue (45 WHO grade 1 and 12 WHO grade 2 and 1 WHO grade 3), acquired from surgery, were immediately transported to the cell culture facility for processing as described below. A portion of these fresh-frozen tissues were used for microarray analysis. Clinical information was collected for each sample, including demographic data, tumor location, treatment options, and prognosis.

### Cell cultures

For monolayer culture, two fresh meningioma tissue samples (named as MEN-117 and MEN-141) were minced and grown in culture medium (Dulbecco’s Modified Eagle Medium, Gibco) with 10% fetal bovine serum and 1% antibiotics (streptomycin and penicillin) and incubated at 37 °C in a humidified atmosphere (5% CO_2_). To prevent loss of character, miRNA transfections of primary cell cultures were done at passage 2. Human meningeal cells (Cat. #1400, ScienCell Laboratories, Carlsbad, California) were cultured according to the provider’s protocol and used as the healthy control.

### Microarray analysis

Tumor tissues were ground with liquid nitrogen, and TRIzol (ThermoFisher Scientific) was added according to the manufacturer’s protocol for total RNA isolation. Whole transcriptome expression profiling was done using Affymetrix Human Gene 2.1 ST Array Strip (Cat no: 902,114, Affymetrix, Santa Clara, CA), which contains over 47,000 transcripts. miRNA microarray analysis was done with the Affymetrix miRNA 4.1 Array Strip (Cat no: 902,404). The chips were used in the GeneAtlas system and the resulting data was analyzed with Transcriptome Analysis Console (TAC) 3 software (Affymetrix).

### miRNA and mRNA expression levels

The expression levels of selected miRNAs in patient samples, meningeal cells, and primary cells grown as a monolayer after anti-miRNA transfection were evaluated with real-time polymerase chain reaction (PCR) using miRNA primers obtained from Exiqon (Vedbæk, Denmark). First, cDNA was synthesized from all miRNA samples according to the manufacturer’s protocol (Exiqon, Cat. No.: 203,300). Synthesized cDNAs were used as templates for gene-expression analysis through real-time PCR, while mRNA levels were measured using Taqman primers (ThermoFisher) after cDNA synthesis. Data was analyzed with the 2^-ΔΔCt^ method. For miRNA normalization, 5S RNA was used. For mRNA normalization, GAPDH was used.

### Optimiziation of anti-miRNA transfection

Cy3 Dye-Labeled Pre-miR Negative Control #1 (Cat: AM17011, ThermoFisher) was used to evaluate the ability of X-tremeGENE siRNA Transfection Reagent (Roche, Cat. No.: 04476093001) to transfect primary meningioma cells under the fluorescent microscope. After validation, anti-miRNA mimics (Ambion Pre-miR miRNA Precursors PM: 114,065) were transfected into cells. To determine the intracellular functionality of anti-miRs, total RNA isolation and miRNA reverse transcription was done and followed by real-time PCR. The targeted miRNA levels were measured 48 h after transfection. The control groups were the X-tremeGENE group, in which only the transfection reagent and medium were delivered to cells, the scrambled miRNA group (Ambion anti-miR miRNA mimics), and the negative control group, which contained only medium.

### Annexin V staining and viability assay

To elucidate the apoptotic effects of hsa-miR-29c-3p and its target PTX3 on primary meningioma cells, annexin V and 7-AAD staining was performed by using apoptosis detection kit I (BD Pharmingen, San Diego, California) 72 h after transfection of anti-miR-29c-3p. Staining was carried out according to the manufacturer’s protocol by using BD FACSAria III cell sorter (BD Biosciences).

Cell viability after anti-miR-29c transfection was assessed at day 3 and day 4 with CellTiter 96 Aqueous One Solution Cell Proliferation Assay (MTS) (Promega, Madison, Wisconsin) according to the manufacturer’s protocol. Scrambled anti-miR was used as a control. Results were obtained by detecting absorbance at a wavelength of 490 nm with the Elisa microplate reader (BioTek, Winooski, Vermont).

### Statistical analysis

The one-way between-subjects ANOVA (unpaired) method was used to evaluate the microarray results. Real-time PCR data was analyzed by using the 2-^ΔΔCt^ method. Spearman’s two-tailed correlation test was used to determine correlations of tumor size and mRNA-miRNA levels, and miRNA and mRNA target levels. For other clinicopathological correlations, the two-tailed chi-square test was used. The calculation and interpretation of *p* values for functions and gene networks (Table 1) was done with a right-tailed Fisher’s exact test. All other statistical analyses were done using student’s t-test. Differences with *p* values of less than 0.05 were considered statistically significant. The high and low miRNA or gene groups of patients were separated according to the median expression level value. Significant outliers were evaluated for each experiment and removed from analysis.

**Table 1:** Number and significance range of molecules participating in relevant pathways and molecular and cellular functions in meningioma. Table derived from data compiled from DAVID bioinformatics database. *P* values were calculated with a right-tailed Fisher’s exact test

Gene Network	*p*-value	Number of Molecules Involved
Inflammatory Response	1.35E-03 – 8.27E-14	182
Cancer	1.28E-03 – 3.20E-11	493
Inflammatory Disease	1.35E-03 – 2.98E-10	161
Cellular Growth	1.23E-03 – 5.70E-18	311
Cell Death-Cell Survival	1.30E-03 – 7.59E-16	265
Cellular Movement	1.34E-03 – 6.11E-15	194
Cellular Development	1.04E-03 – 2.68E-13	257
Cell-Cell Signalling	1.34E-03 – 6.69E-10	157
Immune Cell Traficking	1.06E-03 – 1.95E-09	110

## Results

### Differential expression of miRNAs and mRNAs in meningioma

Fourteen meningioma samples and one healthy human meningeal cell line were used to profile miRNA and gene expression (Additional file 1: Table S1). Of 48,226 genes that were checked for expression, 1257 genes were found to be upregulated and 2496 were found to be downregulated as a result of whole transcriptome microarray (Additional file 2: Figure S1). As a result of miRNA microarray, of 6631 miRNAs checked, 580 genes were found to be upregulated and 311 were found to be downregulated (Additional file 3: Figure S2). The results from both microarrays were combined and analyzed by using TAC (Affymetrix), DAVID, KEGG and Reactome software. To increase confidence, fold change (linear) of less than −4 or fold change (linear) greater than 4 and the ANOVA *p* value (condition pair) less than 0.01 were chosen as selection criteria in both arrays. Deregulated miRNAs and mRNAs were analyzed for their collective effect on important molecular and cellular events (Table 1).

Candidate mRNAs and miRNAs were chosen according to pathways and networks relevant to meningioma, as well as well-matched expression levels of miRNAs and their potential mRNA targets, with the help of web-based databases (mirdb.org/miRDB/, mirtarbase.mbc.nctu.edu.tw, http://targetscan.org/, pictar.mdc-berlin.de, http://www.microrna.org/). The predicted targets were chosen to balance prediction match scores and the relevancy and novelty of the genes in meningioma research (Table 2). The data discussed in this publication have been deposited in NCBI’s Gene Expression Omnibus [4] and are accessible through GEO Series accession number GSE88721 (https://www.ncbi.nlm.nih.gov/geo/query/acc.cgi?acc=GSE88721).

**Table 2:** Selected mRNAs and miRNAs as a result of microarray analysis. Fold change, the matching miRNA for targeting, fold value of miRNA, and information about targeting has already been validated or predicted with software

mRNA	Fold	Targeting miRNA	miRNA Fold	Targeting Status
*PTX3*	-337.5	hsa-miR-29c-3p	21.76	Predicted
*RPL22*	-109.5	hsa-miR-29c-3p	21.76	Validated
*CABIN1*	-8.76	hsa-miR-4492	53.89	Predicted
*RELA*	-7.42	hsa-miR-8089	22.13	Predicted
*SPARCL1*	71.88	N/A		
*TMOD1*	81.71	N/A		

### Confirmation of microarray data with real-time PCR in patient samples

**Fig. 1:**
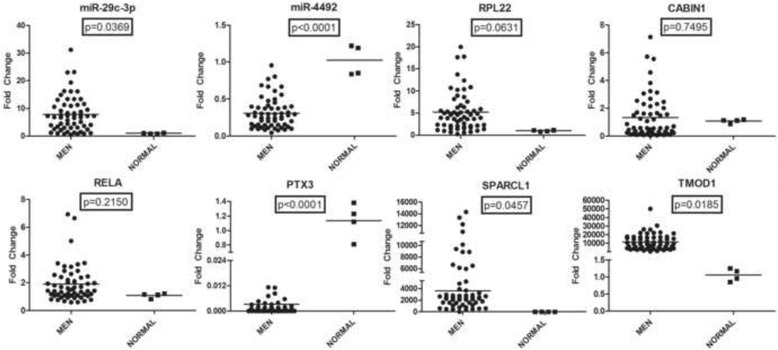
The relative expression levels of selected mRNAs and miRNAs in patient samples as determined by real-time PCR analysis. Each point represents a patient sample or healthy control cell line. *P* values are indicated on each graph

The levels of selected mRNAs and miRNAs in fifty-eight patient samples, were checked. Among the miRNAs, hsa-miR-8089 could not be detected in any of the samples potentially due to a problem in primer design. Although the microarray result suggested that miR-4492-3p levels are higher in meningioma samples than in the healthy control, we have observed the opposite with real-time PCR (Fig. 1). The level of miR-29c-3p in patient samples and healthy controls was consistent with the microarray (Fig. 1).

RPL22, CABIN1, and RELA levels were not significantly different from the controls (Fig. 1) whereas PTX3, SPARCL1, and TMOD1 levels were in synchrony with the microarray results. These are significant as PTX3 was downregulated and SPARCL1 and TMOD1 were upregulated in patient samples compared with controls.

### Anti-miR mimics successfully transfected into cell lines

**Fig. 2:**
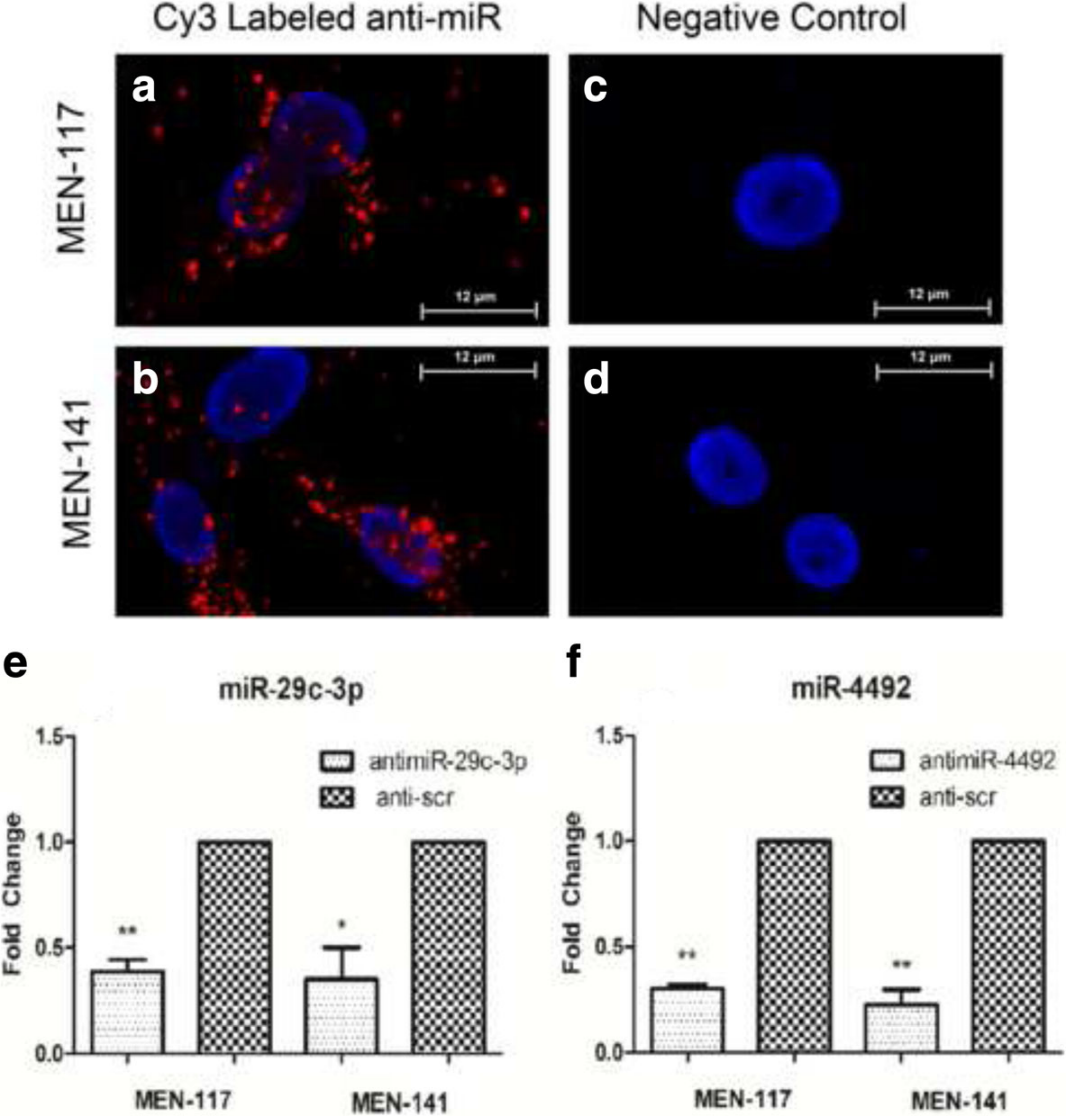
Confirmation of miRNA transfection and anti-miR functionality. Cy3-conjugated scrambled anti-miRNA molecules were transfected into (**a**) MEN-117, (**b**) MEN-117 negative control, (**c**) MEN-141, and (**d**) MEN-141 negative control for direct observation of cellular uptake. Observation was made 8 h after transfection. The red spots represent Cy3-labeled anti-miRNA molecules and the nucleus is stained with DAPI (*blue*). No Cy3 labeled anti-miRNA mimics were transfected to negative controls but only DAPI. Scale bars indicate 12 μm length. Anti-miRNA molecules were transfected into MEN-117 and MEN-141 cells. The ability of anti-miRNA molecules to decrease corresponding miRNA levels: (**e**) The decreased level of miR-29c-3p by anti-miR-29c-3p. **f** The decreased level of miR-4492 by anti-miR-4492. *p < 0.05; **p < 0.01

We assessed the transfection capability of the X-tremeGENE siRNA Transfection Reagent, and the ability of the anti-miR mimics to suppress their target miRNAs in two primary patient-derived meningioma cells. We used Cy3 Labeled Pre-miR Negative Control #1 (ThermoFisher, AM17011) to visualize the presence of anti-miRNA mimic molecules inside the cell 8 h after transfection. Fluorescence microscopy showed that the Cy3-labeled miRNA constructs were successfully transfected into cells (Fig. 2a,b). No fluorescence was detected in the control groups (Fig. 2c,d). Anti-miR mimics were transfected into patient-derived cell lines, and real-time PCR after 48 h revealed that the corresponding anti-miR mimics significantly decreased the level of hsa-miR-29c-3p and hsa-miR-4492-3p (Fig. 2e,f). Levels of hsa-miR-8089 could not be detected.

### Transfection of anti-hsa-miR-29c-3p increased oncosuppressor PTX3

**Fig. 3:**
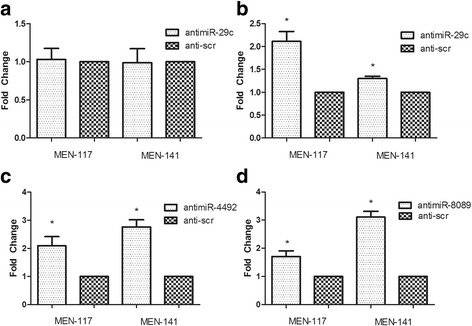
Target mRNA expression levels after administration of corresponding anti-miRNA molecules. Anti-miRNA molecules of miRNAs, miR-29c-3p, miR-4492, and miR-8089, were administered to MEN-117 and MEN-141, and corresponding target mRNA levels were determined after 48 h. **a** RPL22 as the target of miR-29c-3p. **b** PTX3 as the target of miR-29c-3p. **c** CABIN1 as the target of miR-4492. **d** RELA as the target of miR-8089. *Significant changes (*p* < 0.05)

The expression level of validated target RPL22 and predicted target PTX3 was checked 48 h after anti-miR-29c-3p transfection. We did not observe a significant effect on the RPL22 level compared to the control (Fig. 3a). The PTX3 gene expression level increased significantly in primary cell lines MEN-117 and MEN-141, suggesting a regulation of the gene by miR-29c-3p in meningiomas (Fig. 3b). The expression level of the predicted target of hsa-miR-4492, CABIN1, increased both in MEN-141 and MEN-117 cells (Fig. 3c). Although results for miR-8089 expression level confirmation with real-time PCR could not be obtained we decided to carry on with experiments that are not related to measuring the expression level of the miRNA. The level of RELA consistently increased in both MEN-117 and MEN-141 upon transfection with anti-miR-8089 (Fig. 3d).

### Downregulation of hsa-miR-29c-3p decreased cell viability and induced apoptosis in meningioma Cells

**Fig. 4:**
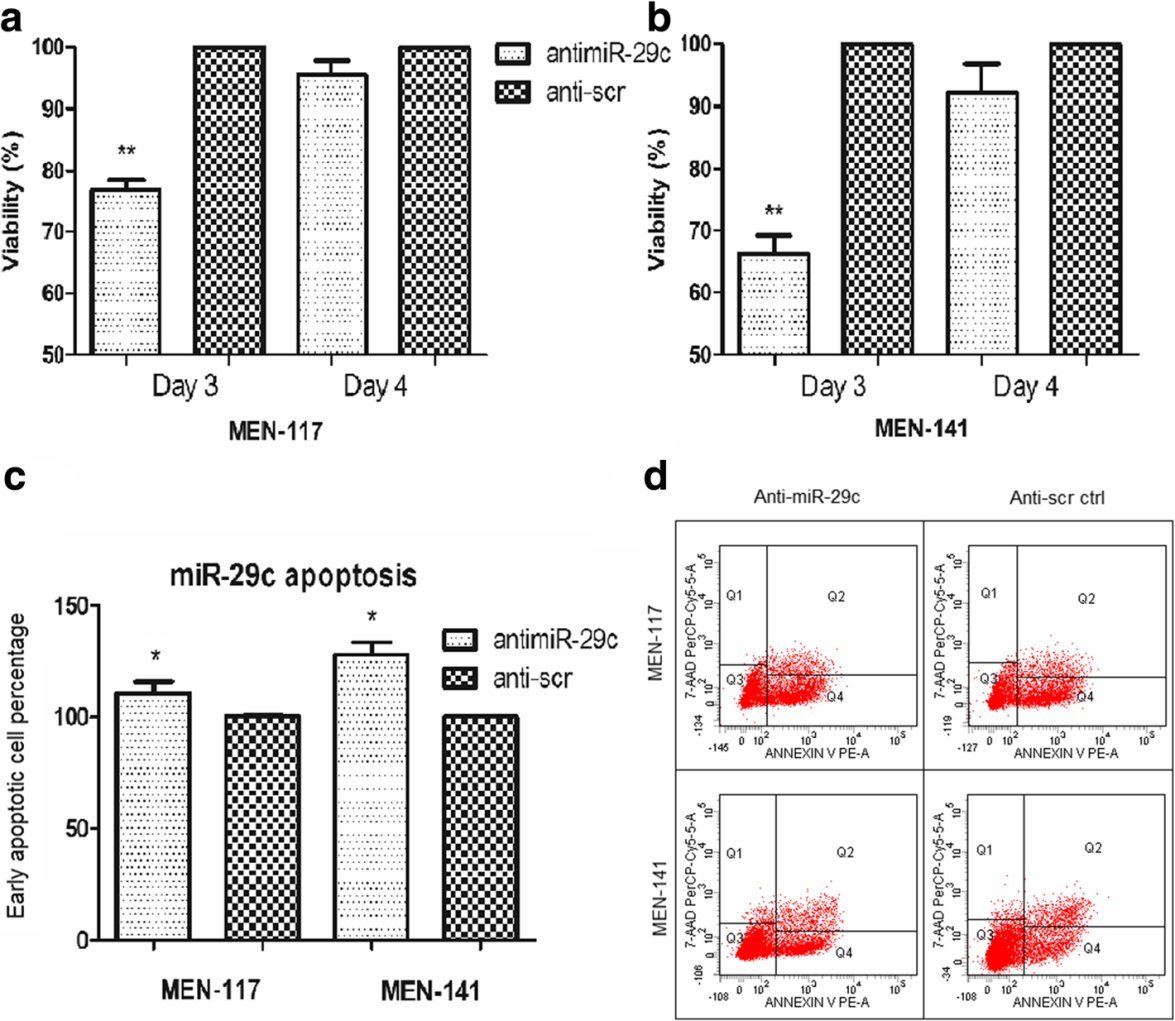
miR-29c-3p has an anti-proliferative and apoptotic effect on meningioma cells. Anti-scr transfected cells were used as the control groups (**a, b**), and viability was measured with an MTS assay after transfection of miR-29c-3p into meningeal cells for 72 and 96 h. **c** Early apoptosis (annexin V + 7-AAD) rate after transfection of miR-29c-3p into meningeal cells for 72 h. **d** Representative images of Annexin-V/7-AAD staining of meningeal cells after miR-29c-3p transfection for 72 h

To observe the viability and percentage of apoptotic cells, anti-miR molecules against miR-29c-3p were transfected into two meningioma primary culture cells, MEN-117 and MEN-141. MTS cell viability analysis resulted in the decrease of viability of anti-miR-29c transfected cells as compared to controls at day 3. This effect was neutralized at day 4 (Fig. 4a,b).

For flow cytometry analysis, the population for apoptotic cells was chosen as the annexin V-positive and 7AAD- negative cells to eliminate any confusion of late apoptotic and necrotic cells, which are positive for annexin V and 7AAD. The results showed that the anti-miR-29c-3p molecules significantly increased apoptosis after 72 h by 10% in MEN-117 and by 28% in MEN-141 when compared with the anti-scr control (Fig. 4c,d).

### Correlation between the level of dysregulated miRNAs and mRNAs with clinicopathological features of meningioma patients

We evaluated the relationships between miR-29c-3p, miR-4492, PTX3, RPL22, CABIN1, RELA, SPARCL1, and TMOD1 levels and the clinicopathological features of meningioma patients including sex, age, tumor grade, tumor volume, calcification, progesterone receptor status, and p53 and Ki67 levels, as well as the correlation between the level of miRNAs and their targets. miR-8089 was excluded since its level of expression could not be determined by real-time PCR. For the chi-square tests, patients were separated into two groups of low and high levels of the corresponding miRNA or mRNA level.

**Fig. 5:**
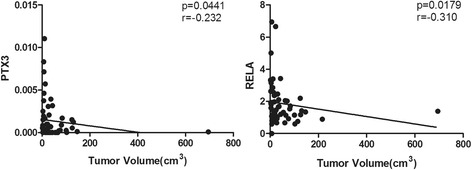
Correlation between the expression level of selected PTX3 and RELA with tumor volume. Selected miRNAs and mRNAs were analyzed with Spearman’s non-parametric correlation test for tumor volume. Significant results are shown here. PTX3 and RELA expressions are indicative of lower tumor volume in the meningioma cohort

Among the clinical characteristics evaluated, miR-29c-3p was negatively correlated with Ki67 index. PTX3, RPL22, CABIN1 and SPARCL1 expression was negatively correlated with progesterone receptor. RELA and TMOD1 were negatively correlated with calcification (Table 3). PTX3 and RELA were negatively correlated with tumor volume in our cohort (Fig. 5).

**Table 3:** Correlation between the expression level of selected miRNAs and mRNAs with clinicopathological features. Selected miRNAs and mRNAs were analyzed with Spearman’s non-parametric correlation test and the chi-square test for age, sex, WHO grade, calcification, progesterone receptor status, p53 status, and Ki67 index. Significant results are shown here

Molecule Name	Clinicopathological Features	High	Low	*Total*	*p-Value*
miR-29c-3p	Ki67 index				
	>7	12	19	31	0.0471
	≤7	17	10	27	
PTX3	Progesterone Receptor				
	Positive	16	25	41	0.0002
	Negative/Focal Positive/Nuclear Positive	11	0	11	
RPL22	Progesterone Receptor				
	Positive	19	22	41	0.0361
	Negative/Focal Positive/Nuclear Positive	9	2	11	
CABIN1	Progesterone Receptor				
	Positive	14	27	41	0.0004
	Negative/Focal Positive/Nuclear Positive	10	1	11	
SPARCL1	Progesterone Receptor				
	Positive	16	25	41	0.0022
	Negative/Focal Positive/Nuclear Positive	10	1	11	
RELA	Calcification				
	Positive	17	25	42	0.0447
	Negative	10	4	14	
TMOD1	Calcification				
	Positive	17	25	42	0.0447
	Negative	10	4	14

## Discussion

Since the genomic revolution began, our knowledge about cancers has rapidly improved, leading to the discovery of molecular markers that predict outcomes and help define the best choice for treatment. In the near future, molecular classification and, consequently, personalized therapy is the ultimate goal of treatment for any tumor. Studies using microarray analysis can reveal gene networks that relate to treatment response, clinical outcome, and clinical progression.

Fifteen different subtypes of meningioma have been identified by the World Health Organization (WHO). These subtypes are further classified into three categories: benign (80%), atypical (15%–20%), and malignant (1%–3%) [5]. An atypical meningioma is diagnosed by observing necrosis, sheeting, prominent nucleoli, cellularity, and cell size along with the recent criterion of brain invasion [6].

MicroRNAs (miRNAs) are small, non-coding RNA molecules that are about 22 nucleotides long. There is growing interest in miRNAs with respect to their role in the initiation and progression of cancer, but studies of the miRNA profile of meningiomas are limited in number. In one study, 60 sporadic meningiomas and three healthy arachnoidal tissue samples were used in a whole genome array to find that miR-200a down regulation was associated with tumor growth, epithelial-to-mesenchymal transition status, and Wnt signaling [7]. miR-145, which decreased proliferation and induced apoptosis in vitro and in vivo, was downregulated in atypical and anaplastic meningiomas when compared with benign meningiomas and this molecule [8].

Previous studies using microarrays led to the discovery of important dysregulated mRNA molecules and altered pathways, such as IGF2, Wnt, PI3K, MAPK, MMP12, and TGF-β [9–12]. But few studies of the miRNA profiling of meningiomas have been conducted. A number of miRNAs have been found to be dysregulated. These include miR-145, let-7d, miR-335, miR-98, miR-181a, miR-200a, miR-373*, miR-575, miR-335, miR-96-5p, miR-190a, miR-29c-3p, and miR-219-5p [7, 8, 13, 14]. But only two of these studies incorporated microarray data. In addition, no common miRNA has been identified in any two of these studies, reflecting the lack of well-designed studies in this field of research.

Our microarray data defined three miRNAs that can play a role in gene networks and that are potentially important for meningioma initiation and progression. To the best of our knowledge, there is no previous integrated miRNA-mRNA study of meningiomas that uses microarray data. In addition, ours is the first study in which a miRNA and mRNA expression profiles have been observed in the same samples. Combining the data from the two most up-to-date arrays provided us with valuable information on potential gene networks in the disease about inflammatory responses, cancer, cellular growth and survival, cellular movement and development, cell-to-cell signaling and immune cell trafficking. A limitation to our study is the usage of one healthy human cell line due to unavailability of more commercial cell lines and ethical difficulty in acquiring healthy meningeal tissue from patients du to ethical responsibilities. In further studies the number of healthy controls should be increased and should not be limited to only cell lines but also healthy meningeal tissue.

In this study, we found that miR-29c-3p is upregulated in meningiomas, whereas its predicted target PTX3 is downregulated. Inhibiting miR-29c-3p has increased the expression level of PTX3 in primary meningioma cells, indicating a potential targeting of PTX3 by miR-29c. miR-29c-3p was found to be downregulated when compared with adjacent tissue in meningioma. In the same study, lower miR-29c was associated with advanced clinical stages of meningioma which is in synchrony with our finding that lower miR-29c is associated with a higher ki67 index [14]. The miR-29 family members miR-29a, miR-29b, and miR-29c have diverse roles in cancer [15] by inhibiting tumorigenesis [16], promoting cancer cell apoptosis [17], and suppressing cell proliferation [18]. On the other hand, the miR-29 family can induce an epithelial-to-mesenchymal transition acting as drivers of tumor growth and metastasis [19]. In our study, the downregulation of miR-29c decreased cell viability and increased apoptosis.

PTX3 plays a role in inflammation, both endogenously and exogenously, with dual effects on the process [20, 21]. PTX3 is considered a tumor suppressor gene that plays a role in tumor-promoting inflammation in cancer [22]. Our data show that the tumor suppressor PTX3 is constitutively downregulated in meningiomas. PTX3 level was negatively correlated with tumor volume and progesterone receptor level in our cohort which supports the argument that the gene can act as a tumor suppressor for meningioma. Progesterone level relates to the WHO grade and Ki67 status of meningiomas, as previously reported [23]. Furthermore, our microarray data show the altered expression of hundreds of molecules that take part in inflammatory pathways. These findings suggest that the emerging cancer hallmark of tumor-promoting inflammation is potentially a driving force in the initiation and progression of meningiomas with PTX3 potentially taking part in the process.

We also assessed relationship between the selected miRNAs and mRNAs and clinicopathological features. High TMOD1 and RELA levels were associated with low calcification in our patient group. Calcification is considered predictive of outcome in meningioma patients. The level of calcification in tumors seen on magnetic resonance images is used to determine the treatment strategy for the tumor. Calcification in meningiomas is associated with a low growth rate, suggesting a conservative treatment option [24]. RELA level is also associated with a lower tumor volume in our cohort. RPL22, CABIN1 and SPARCL1 levels were also negatively correlated with the progesterone receptor level, a relation similar to that of PTX3.

The level of miR-4492 was found to be upregulated in our microarray data. However real-time PCR confirmation tests revealed that this miRNAs was significantly downregulated not only in the group that microarray analysis was conducted but also in our extended cohort. This is probably due to an error in the microarray design for the particular miRNA which is relatively recently discovered. This result shows that microarray assessments may not always be indicative of the level of expression and confirmation with real-time PCR is crucial to detect the expression level.

## Conclusions

Our study presents valuable integrated data about mRNA and miRNA expression in meningioma samples. Markers that can play a role in meningioma pathophysiology and tumor-promoting inflammation have been determined, and the results reveal that the relationship between miR-29c-3p and PTX3 can be one of the driving forces in meningioma pathology. Further studies of these gene networks can produce translational information, leading to a better understanding of the initiation and progression of meningiomas and perhaps introducing alternative treatment approaches for the disease.

## Additional files

Additional file 1: Table S1. An overview of the patient cohort. Tumor location and stage definition of patient samples. Median age of patients was 55.5 and age range was 15-89. (DOCX 17 kb)

Additional file 2: Figure S1. Array study report for gene expression microarray: Summary of the gene expression array results, scatter plot and volcano plot generated by TAC software (Affymetrix). (TIFF 355 kb)

Additional file 3: Figure S2. Array study report for miRNA microarray: Summary of the miRNA array results, scatter plot and volcano plot generated by TAC software (Affymetrix). (TIFF 276 kb)

## Abbreviations

WHO: World health organization
miRNA: micro RNA
PCR: Polymerase Chain Reaction
ANOVA: Analysis of variance
