# Supplementary material for: Simultaneous analysis of miRNA-mRNA in human meningiomas by integrating transcriptome: A relationship between PTX3 and miR-29c
Source: BMC Cancer. 2017 Mar 21;17:207. doi: 10.1186/s12885-017-3198-4 (PMC5361823; doi:10.1186/s12885-017-3198-4)
Supplement: Supplementary file 1 — An overview of the patient cohort. Tumor location and stage definition of patient samples. Median age of patients was 55.5 and age range was 15-89. (DOCX 17 kb) [file 12885_2017_3198_MOESM1_ESM.docx]

**Table S1:** **An overview of the patient cohort**. Tumor location and stage definition of patient samples. Median age of patients was 55.5 and age range was 15-89.

| **Location** | **Stage** |  |  |
| --- | --- | --- | --- |
| Olfactor Groove | Meningoteliomateus (WHO grade I) |  |  |
| Sphennopetroclival | Meningotelial (WHO Grade I ) |  |  |
| Parietal Convexity | Atypical (WHO Grade II) |  |  |
| Petrous | Meningoteliomateus (WHO grade I) |  |  |
| Petroclival | Meningoteliomateus (WHO grade I) |  |  |
| Parietal Parasagital | Transitional (WHO Grade I) |  |  |
| Left cavernous Sinus | Atypical (WHO Grade II) |  |  |
| Tuberculum Sella | Meningoteliomateus (WHO grade I) |  |  |
| Right frontobasal | Meningoteliomateus (WHO grade I) |  |  |
| Left sphenoid wing | Meningoteliomateus (WHO grade I) |  |  |
| Right parietal c | Fibroblastic (WHO grade I) |  |  |
| Left Clinoidal | Angiomateus and meningoteliomateus (WHO grade I) |  |  |
| Left Parietal Parasagittal | Fibroblastic (WHO grade I) |  |  |
| Tuberculum Sella meningioma | Meningoteliomateus (WHO grade I) |  |  |
| Foramen magnum | Kordoid Meningioma (WHO Grade II) |  |  |
| Clinoidal | Meningothelial (WHO grade I) |  |  |
| Parasagital | Meningothelial (WHO grade I) |  |  |
| Clinoidal | Angiomateus (WHO grade I) |  |  |
| Petreus | Fibroblastic (WHO grade I) |  |  |
| Multifocal | Meningothelial (WHO grade I) |  |  |
| Clinoidal | Meningothelial (WHO grade I) |  |  |
| Clivus | Meningothelial (WHO grade I) |  |  |
| Convexity | Transitional (WHO grade I) |  |  |
| Sylvian | Meningothelial (WHO grade I) |  |  |
| Petroclival | Meningothelial (WHO grade I) |  |  |
| Falx | Transitional (WHO grade I) |  |  |
| Falx | Atypical (WHO Grade II) |  |  |
| Petroclival | Transitional (WHO grade I) |  |  |
| Parasagital | Transitional (WHO grade I) |  |  |
| Parasagital | Transitional (WHO grade I) |  |  |
| Falx | Meningothelial (WHO grade I) |  |  |
| Petroclival | Meningothelial (WHO grade I) |  |  |
| Sphenoid Wing | Atypical (WHO Grade II) |  |  |
| Convexity | Atypical (WHO Grade II) |  |  |
| Convexity | Meningothelial (WHO grade I) |  |  |
| Falx | Atypical (WHO Grade II) |  |  |
| Falx | Angiomateus (WHO grade I) |  |  |
| Clinoidal | Meningothelial (WHO grade I) |  |  |
| Petreus | Meningothelial (WHO grade I) |  |  |
| Convexity | Meningothelial (WHO grade I) |  |  |
| Petrocliva | Sectretuary (WHO grade I) |  |  |
| Sphenoid Wing | Meningothelial (WHO grade I) |  |  |
| Petroclival | Psammomateus and Meningothelial (WHO grade I) |  |  |
| Scull base | Atypical (WHO Grade II) |  |  |
| Convexity | Angiomateus (WHO grade I) |  |  |
| Foramen magnum | Psammomateus and Meningothelial (WHO grade I) |  |  |
| Sinus Rectus | Transitional (WHO grade I) |  |  |
| Tentorium | Meningothelial (WHO grade I) |  |  |
| Tentorium | Atypical (WHO Grade II) |  |  |
| Lateral Ventricule | Transitional (WHO grade I) |  |  |
| Convexity | Atypical (WHO Grade II) |  |  |
| Foramen magnum | Meningothelial (WHO grade I) |  |  |
| Convexity | Fibroblastic (WHO grade I) |  |  |
| Foramen magnum | Meningioma |  |  |
| Multifocal convexity | Kordoid Meningioma (WHO Grade II) |  |  |
| Convexity | Transitional (WHO grade I) |  |  |
| Falx | Meningothelial (WHO grade I) |  |  |
| Falx | Malign Meningioma (WHO Grade III) |  |  |
