# Supplementary figures and images for: Simultaneous analysis of miRNA-mRNA in human meningiomas by integrating transcriptome: A relationship between PTX3 and miR-29c
Source: BMC Cancer. 2017 Mar 21;17:207. doi: 10.1186/s12885-017-3198-4 (PMC5361823; doi:10.1186/s12885-017-3198-4)

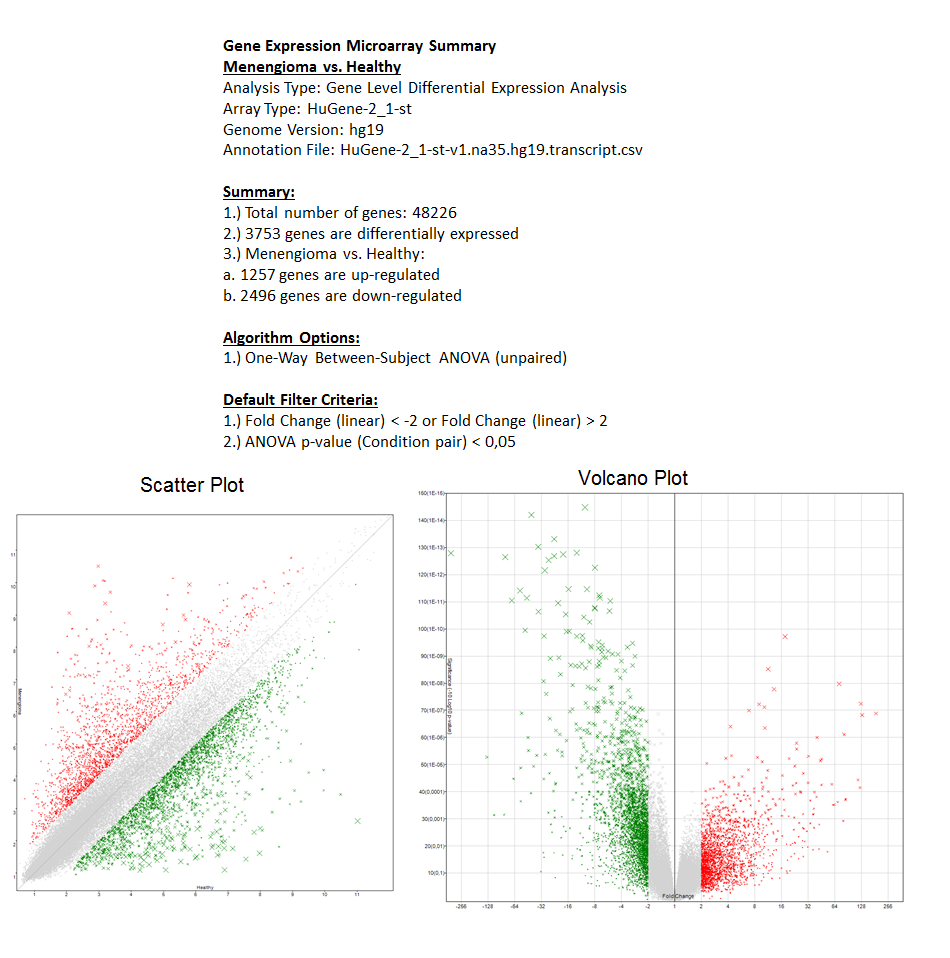

Supplement: Supplementary file 2 — Array study report for gene expression microarray: Summary of the gene expression array results, scatter plot and volcano plot generated by TAC software (Affymetrix). (TIFF 355 kb) [file 12885_2017_3198_MOESM2_ESM.tif]

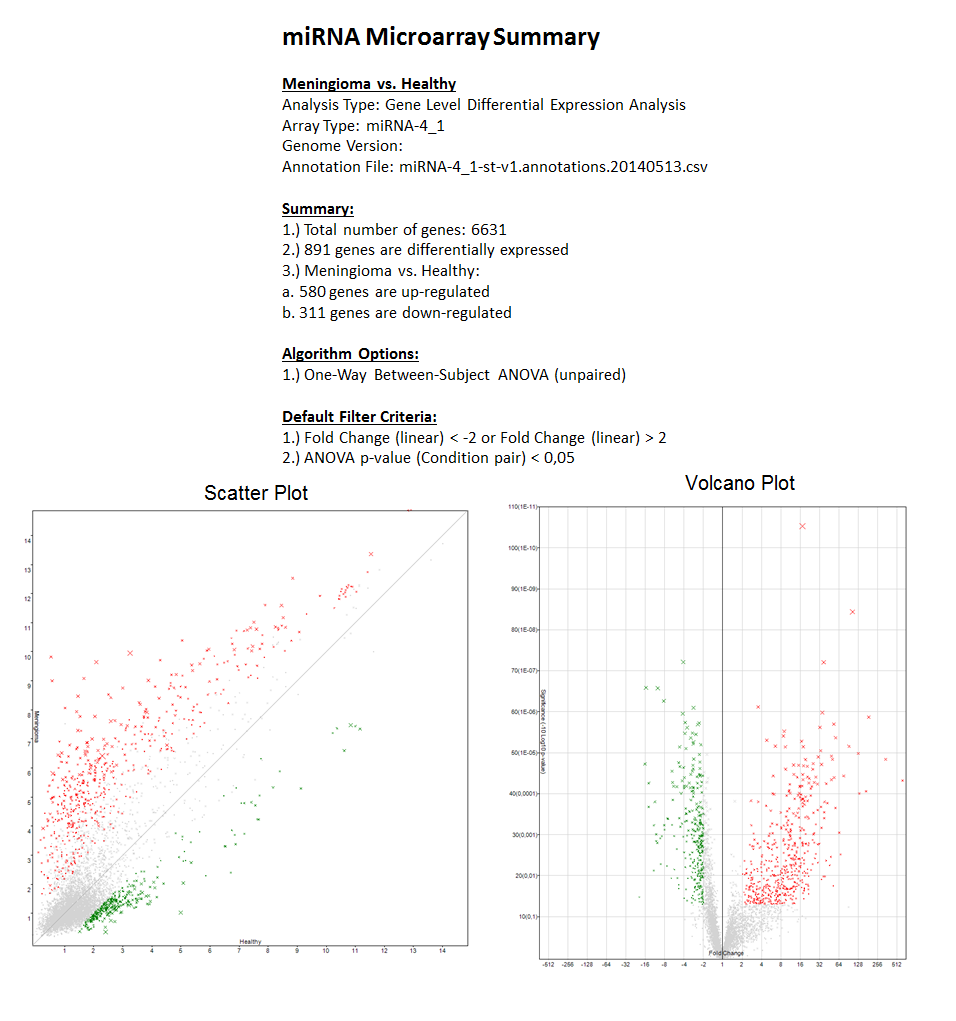

Supplement: Supplementary file 3 — Array study report for miRNA microarray: Summary of the miRNA array results, scatter plot and volcano plot generated by TAC software (Affymetrix). (TIFF 276 kb) [file 12885_2017_3198_MOESM3_ESM.tif]
